# Effect of meteorological factors on *Culex* mosquitoes in Singapore: a time series analysis

**DOI:** 10.1007/s00484-020-02059-9

**Published:** 2021-01-10

**Authors:** Annabel Seah, Joel Aik, Lee-Ching Ng

**Affiliations:** 1 Environmental Health Institute, National Environment Agency, 40 Scotts Road, Environment Building, #13-00, Singapore, 228231 Singapore; 2 School of Public Health and Community Medicine, Faculty of Medicine, University of New South Wales, Kensington, New South Wales Australia

**Keywords:** *Culex*, Weather, Climate change

## Abstract

*Culex* mosquitoes transmit West Nile virus (WNV). We examined the weather dependence of adult *Culex* activity. Maximum temperature and absolute humidity were positively associated with adult *Culex* activity. Our findings support the use of weather data in timing WNV vector control measures.

**Supplementary Information:**

The online version contains supplementary material available at 10.1007/s00484-020-02059-9.

The *Culex* mosquito is a vector of the West Nile virus (WNV) (Paz & Semenza, 2013). Migratory birds which are viral reservoirs travel along the East Asia Australasian Flyway, through WNV-endemic USA, before stopping over in Singapore (Yap et al. 2019), and may import WNV. Elevated global temperatures, as a result of climate change, accelerate mosquito development, thus resulting in changes in the transmission pattern of *Culex* transmitted viruses (Paz and Semenza 2013). While other studies have examined the effect of short-term climate variations on the adult *Culex* population in temperate (Paz and Semenza 2013), continental (Karki et al. 2016) and subtropical (Rueda et al. 1990) climate settings, this has not been investigated in the tropics.

We obtained data on ambient temperature, cumulative rainfall and absolute humidity (AH) from weather stations located across Singapore. We examined the short-term impact of these climate conditions on the adult *Culex* index, using data obtained from the national Gravitrap surveillance program (Lee et al. 2013) on the rate of trapped adult female *Culex quinquefasciatus*—the most common *Culex* species in Singapore—which is defined as

$$ Culex\ index=\frac{Total\ number\ of\ adult\ female\ mosquitoes}{Total\ number\ of\ Gravitraps} $$

from epidemiologic week (E-week) 44 of 2017 to E-week 7 of 2020.

Since the *Culex* mosquito lifecycle duration (7–10 days) (Centers for Disease Control and Prevention 2019) and adult lifespan (18–23 days) (Andreadis et al. 2014) total to 25–33 days or around 5 weeks at maximum, we included immediate and delayed effects of all climatological variations up to this duration. This was similar to the lag duration reported in another study (Karki et al. 2016). We used a distributed lag non-linear model in the “dlnm” package (version 2.3.9) in R software (version 3.5.2) to account for delayed, non-linear effects of weather on adult *Culex* index,*Y*_*t*_ in week *t*, as shown in Eq. 1:

1 $$ {\displaystyle \begin{array}{c}\ \\ {}{\mu}_t={\beta}_0+ ns\left(t, df\right)+S\left({x}_{j,t},{\varphi}_j,\tau\ \right)+{\beta}_1\sum \limits_{l=1}^{l=L}{res}_l\ \end{array}} $$

where *μ*_*t*_ is the expected adult *Culex* index in week *t* and *β*_0_ represents the intercept. We accounted for trend and seasonality in *Y*_*t*_ using natural cubic splines (*ns*, *df*), with 4 *df*(degrees of freedom) per year. *ns* functions with 3 *df* are used to describe the smoothed *S*(*x*_*j*, *t*_, *φ*_*j*_,  *τ*) relationship between *μ*_*t*_ and “cross-basis” matrices of each weather variable, *x*_*j*_, for up to τ = 5-week lag respectively. Coefficient vector *φ*_*j*_ represents changes in adult *Culex* index for a unit change in *x*_*j*_ weather parameter. We added lags of deviance residuals *res*_*l*_ to account for serial correlation.

The U-shaped non-linear associations between the change in adult *Culex* index and MaxT demonstrate a less pronounced increase in adult *Culex* index as MaxT increases up to 30.5 °C but becomes more pronounced as MaxT increases beyond 31.9 °C (Fig. 1a). Increasing temperature accelerates larvae development, leading to subsequent increases in adult *Culex* abundance. However, this increase in adult *Culex* abundance plateaus at higher MaxT as increased thermal stress reduces adult survival. At higher MaxT threshold, another biological mechanism may be predominant. In Singapore, dry weather with higher temperatures are associated with excessive leaf shedding, resulting in a leaf litter build-up in drains (Ee 2014). Upon decomposition, the leaf litter habitat provides organic nutrients for the mosquito larvae (Noori et al. 2015), resulting in increased mosquito abundance.

**Fig. 1:**
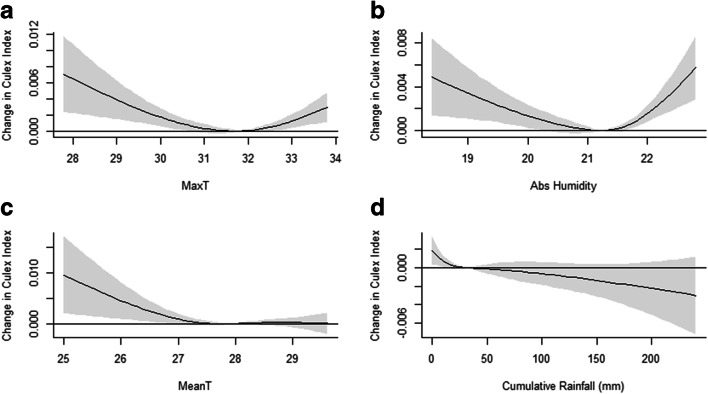
Exposure-response curve showing overall cumulative effect on adult *Culex* activity. Overall cumulative effect of weekly **a** MaxT, mean-centred at 31.8 °C; **b** absolute humidity mean-centred at 21.2 g/m^3^, **c** MeanT, mean-centred at 27.9 °C; and **d** cumulative rainfall, mean-centred at 32.1 mm. Shaded grey areas indicate 95% confidence intervals (CIs)

Similarly, the increase in adult *Culex* index becomes less pronounced as AH increases up to 20.2 g/m^3^ but becomes more pronounced as AH increases beyond 21.2 g/m^3^ (Fig. 1b). We found the correlation between MaxT and AH to be weak and statistically insignificant (*r* = 0.064, *p* = 0.489). Therefore, despite having similar U-shaped linear association as MaxT, the association observed between AH and adult *Culex* index is independent of MaxT. Instead, it could be due to an interplay of less shedding of dry leaves at lower AH levels, resulting in reduced availability of viable breeding sites, but increased adult mosquito survival at higher AH thresholds as the higher atmospheric moisture content reduces egg and adult desiccation stress (Benoit et al. 2010).

The increase in adult *Culex* index becomes less pronounced as mean temperature increases up to 26.8 °C—similar to the results obtained for MaxT of this temperature range (Fig. 1c). Rainfall was negatively associated with the adult *Culex* index (Fig. 1d). While this relationship was statistically insignificant, it is consistent with other studies (Karki et al. 2016; Paz and Semenza 2013) and is plausible as heavy rainfall flushes *Culex* larvae from their unsheltered habitats (Karki et al. 2016), resulting in reduced survival to adulthood.

Our study provides evidence to support the relationship between short-term weather variations and adult *Culex* activity. With global warming, hotter and more humid weeks favouring adult *Culex* activity may be expected. Public health authorities seeking to reduce the risk of WNV transmission in tropical urban settings could time their vector control measures in anticipation of weather driven increases in adult *Culex* activity.

## Supplementary information

ESM 1 (PDF 222 kb)

## Data Availability

The weather data presented in this study are available upon reasonable request from the Meteorological Services Singapore (MSS) of the NEA (email: Contact_NEA@nea.gov.sg). The *Culex* data presented in this study are available upon reasonable request from the Environmental Public Health Operations Department (EPHOD) of the NEA (email: Contact_NEA@nea.gov.sg).

## Abbreviations

WNV: West Nile virus
AH: Absolute humidity
MeanT: Mean temperature
MaxT: Maximum temperature
