# Supplementary material for: Effect of meteorological factors on Culex mosquitoes in Singapore: a time series analysis
Source: Int J Biometeorol. 2021 Jan 10;65(6):963–5. doi: 10.1007/s00484-020-02059-9 (PMC8149361; doi:10.1007/s00484-020-02059-9)
Supplement: Supplementary file 1 — (PDF 222 kb) [file 484_2020_2059_MOESM1_ESM.pdf]

Supplemental Material

The influence of temperature and humidity on the abundance of *Culex quinquefasciatus* mosquitoes in Singapore

Authors

Annabel Seah[1], Joel Aik[1,2*] and Lee-Ching Ng[1]

[1] Environmental Health Institute, National Environment Agency (40 Scotts Road, Environment Building, #13-00, Singapore 228231)

[2] School of Public Health and Community Medicine, Faculty of Medicine, University of New South Wales, New South Wales, Australia

*Corresponding author

Email addresses of authors:

Annabel Seah: annabel_seah@nea.gov.sg

Joel Aik: joel_aik@nea.gov.sg

Lee-Ching Ng: ng_lee_ching@nea.gov.sg

Table 1: Weekly measures of trapped adult female *Culex quinquefasciatus* and climate conditions in Singapore, from epidemiologic week (E-week) 44 of 2017 to E-week 7 of 2020. IQR refers to the interquartile range, 1st – 3rd Quartile

	Mean (SD)	Median	IQR	Minimum	Maximum
**Adult female *Culex quinquefasciatus***	108 (71)	81	54-142	31	301
**Mean Temperature (°C)**	28.0 (0.9)	28.0	27.4-28.6	25.0	29.7
**Maximum Temperature (°C)**	31.9 (1.0)	32.0	31.3-32.5	27.7	33.9
**Absolute Humidity (g/m^3)**	21.2 (0.8)	21.3	20.7-21.8	18.3	22.9
**Cumulative Rainfall (mm)**	32.1 (41.1)	18.9	2.9-47.8	0.0	244.0
**Gravitraps**	52333 (2282)	50919	50535-53822	48874	56369
